# Pre-clinical evaluation of Minnelide as a therapy for acute myeloid leukemia

**DOI:** 10.1186/s12967-019-1901-8

**Published:** 2019-05-20

**Authors:** Bhuwan Giri, Vineet K. Gupta, Brianna Yaffe, Shrey Modi, Pooja Roy, Vrishketan Sethi, Shweta P. Lavania, Selwyn M. Vickers, Vikas Dudeja, Sulagna Banerjee, Justin Watts, Ashok Saluja

**Affiliations:** Sylvester Comprehensive Cancer Center and DeWitt Daughtry Family Department of Surgery, University of Miami, 460C CRB Research Building, 1140 NW 14th St, Miami, FL 33136 USA

**Keywords:** Minnelide, Acute myeloid leukemia, c-Myc, AML, Triptolide

## Abstract

**Background:**

There is an urgent need for novel and effective treatment options for acute myeloid leukemia (AML). Triptolide, a diterpenoid tri-epoxide compound isolated from the herb *Tripterygium wilfordii* and its water-soluble pro-drug-Minnelide have shown promising anti-cancer activity. A recent clinical trial for patients with solid tumors confirmed the safety and efficacy at biologically equivalent doses of 0.2 mg/kg/day and lower.

**Methods:**

Cell viability of multiple AML cell lines as well as patient apheresis samples were evaluated by 3-(4,5-dimethylthiazol-2-yl)-2,5-diphenyl tetrazolium bromide (MTT) based assay. Apoptosis was evaluated by estimating the amount of cleaved caspase. AML cell line (THP1-Luc) was implanted in immunocompromised mice and treated with indicated doses of Minnelide. Leukemic burden before and after treatment was evaluated by imaging in an In Vivo Imaging System (IVIS).

**Results:**

In the current study, we show that Minnelide, at doses below maximum tolerated dose (MTD) demonstrates leukemic clearance of both primary AML blasts and luciferase expressing THP-1 cells in mice. In vitro, multiple primary AML apheresis samples and AML cell lines (THP-1, KG1, Kasumi-1, HL-60) were sensitive to triptolide mediated cell death and apoptosis in low doses. Treatment with triptolide led to a significant decrease in the colony forming ability of AML cell lines as well as in the expression of stem cell markers. Additionally, it resulted in the cell cycle arrest in the G1/S phase with significant downregulation of c-Myc, a major transcriptional regulator mediating cancer cell growth and stemness.

**Conclusion:**

Our results suggest that Minnelide, with confirmed safety and activity in the clinic, exerts a potent anti-leukemic effect in multiple models of AML at doses easily achievable in patients.

**Electronic supplementary material:**

The online version of this article (10.1186/s12967-019-1901-8) contains supplementary material, which is available to authorized users.

## Background

In 2018, almost 21,000 new cases of acute myeloid leukemia (AML) will be diagnosed with an estimated 10,500 deaths [1]. In the absence of intensive chemotherapy, median survival of patients with AML is a mere 5–10 months [2]. The general treatment guidelines for AML have not changed in the last 40 years [3] and consists of high dose infusion of cytarabine in combination with an anthracycline (doxorubicin or daunorubicin). As such, this aggressive chemotherapy regimen is poorly tolerated and comes with significant side effects [4]. Newer targeted therapies have shown promise but act only on a small subset of patients [5, 6]. Furthermore AML typically affects the elderly [7] which tolerate the aggressive chemotherapy regimen poorly. Also, even with this aggressive toxic chemotherapy regimen, only 15% of patients older than 65 years of age will achieve complete remission [3]. As such, novel therapy that is both well tolerated and effective is the need of the hour.

Previous studies from our laboratory show that triptolide, a diterpene tri-epoxide, and its water soluble pro-drug, Minnelide, are effective against pre-clinical models of multiple cancers [8–11]. Subsequently a Phase I clinical trial for Minnelide showed promising efficacy and tolerability at a dose of 0.67 mg/m^2^/day in patients with advanced gastro-intestinal (GI) malignancies [12]. This translates to an equivalent mouse dose of ~ 0.21 mg/kg/day [13]. In the current study we show that Minnelide is effective against AML at a very low dose (well within the dose range tolerated by patients) both in vivo and in vitro. At mechanistic level we demonstrate that triptolide decreases stemness in AML cancer cells and mediates apoptotic cell death via the Myc pathway.

## Results

### Minnelide reduces tumor burden and halts progression in multiple models of acute myeloid leukemia

**Fig. 1:**
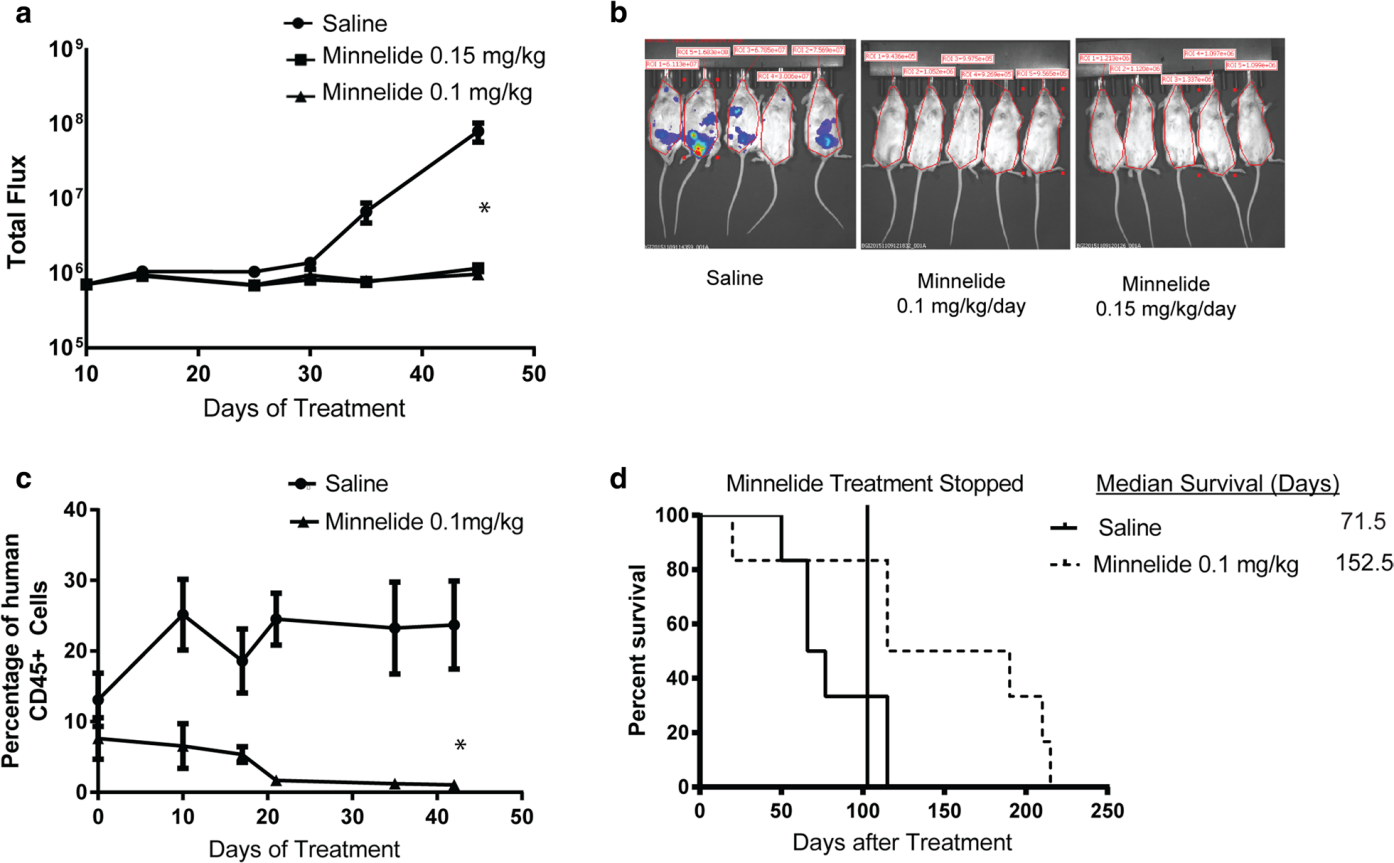
**a** Treatment with Minnelide at a dose of 0.15 mg/kg and 0.1 mg/kg significantly decreased leukemic burden in cancer bearing mice. **b** The total burden of disease was calculated using bioluminescence with the In Vivo Imaging System (IVIS) measuring total flux from a region of interest (ROI) drawn over the entire animal. Total flux is calculated as the radiance (photons/s) in each pixel summed or integrated over the ROI area (cm^2^) × 4π. **c** AML blasts derived from patient leukepheresis was implanted into NRGS mice after sub lethal irradiation and treatment with Minnelide was started at a dose of 0.1 mg/kg/day after confirming engraftment via flow cytometry. Antibody against human CD45 was used to quantify disease burden. Treatment with Minnelide slowed the progression of AML and decreased the tumor burden at endpoint. **d** Even when Minnelide treatment was discontinued at 100 days, there was an improvement in median survival of mice from 71 to 150 days. n = 4–5, *p < 0.05 when compared to saline treated animals, data shows mean ± SEM

To generate an animal model of acute myeloid leukemia, immunodeficient female NRG-SGM3 (NRGS) mice were used. These mice are NOD.Rag1−/−;γcnull (NRG) animals expressing human interleukin-3 (IL-3), human granulocyte/macrophage-stimulating factor (GM-CSF) and human Steel factor (SF) from the SGM3 (3GS) triple co-injected transgenes. The mice were irradiated with 250 cGy, to facilitate engraftment, and 2 × 10^6^ THP-1 AML cells with Luciferase reporter gene were injected via tail vein. These mice were serially followed by bioluminescence imaging to evaluate progression of disease. As seen in Fig. 1a, saline treated control mice show progressive disease and are moribund within 45 days of cancer implantation. Upon treatment with Minnelide at 0.15 mg/kg/day and 0.1 mg/kg/day, progression of AML was dramatically slowed as measured by bioluminescence imaging (Fig. 1a). This is represented by significantly decreased tumor burden in the Minnelide group, as quantified by IVIS and is decreased to baseline levels of bio-luminescence seen in naïve NRGS mice (Fig. 1b and Additional file 1: Figure S1). These results show that Minnelide (at a dose that is known to be clinically well tolerated in patients) is effective in reducing the burden of AML.

In a separate model, leukemic blast cells derived from a patient with AML were implanted in NRGS mice following sub-lethal irradiation. In this model, Minnelide at a dose of 0.1 mg/kg/day showed remarkable activity in reducing blast cell count in peripheral circulation measured by an antibody specific to human CD45 (Fig. 1c). Untreated mice succumbed to progressive disease with a median survival of 71.5 days while Minnelide treated mice had significantly increased survival. To understand if effect of Minnelide are transient or sustained, we saw that withdrawal of Minnelide at 100 days of treatment led to prolonged efficacy with median survival reaching 152.5 days (Fig. 1d). These results show that Minnelide is effective in multiple models of AML. To assess toxicity and evaluate the effect on normal hematopoiesis, we treated disease free C57B6 mice with Minnelide for 14 days and analyzed the number and proportion of normal neutrophils from peripheral circulation as well as their pre-cursors from bone marrow. We saw that neutrophils in spleen, bone marrow and blood as well as hematopoietic stem cells showed no significant changes with Minnelide treatment (Additional file 2: Figure S2).

### Triptolide, the active metabolite of Minnelide, effectively kills AML cells in vitro by apoptosis

**Fig. 2:**
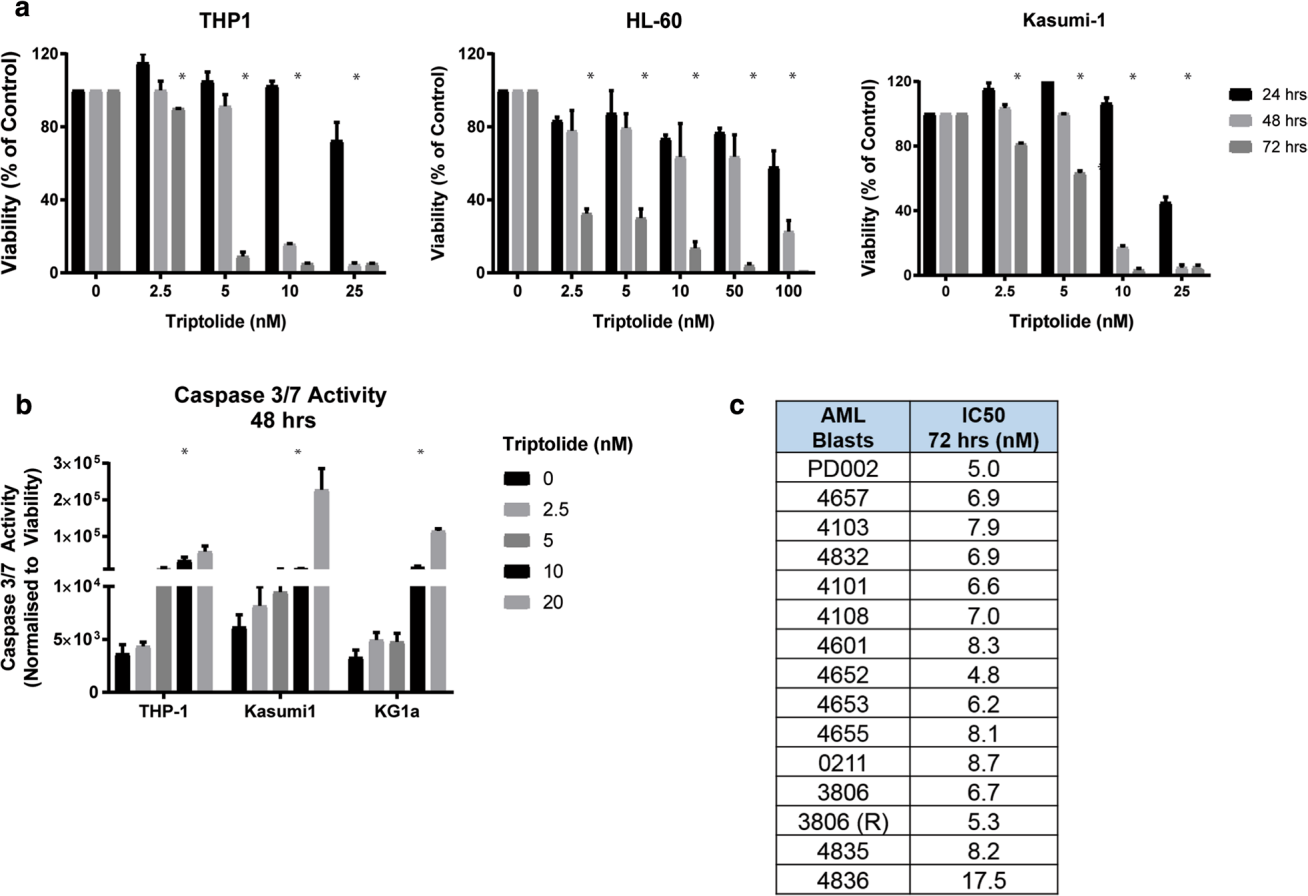
**a** AML cell lines THP-1, HL-60 and Kasumi-1 were treated with increasing concentrations of triptolide and cell viability was evaluated using ATP based luciferase assay. Treatment with triptolide decreased cell proliferation in all cell lines at low nano-molar doses. **b** This decrease in viability corresponded to an increase in apoptotic cell death as measured by the activity of caspase 3. **c** In patient blast samples, triptolide caused cell death with an IC-50 in the range of 4 nm to 17 nM, n = 3, *p < 0.05 when compared to untreated cells, data shows mean ± SD

We used in vitro studies to evaluate the mechanism by which Minnelide kills AML cancer cells. Minnelide is converted to its active form triptolide when it is administered in vivo [14] thus we used triptolide for our in vitro studies. AML cell lines (THP-1, Kasumi-1, HL-60) treated with triptolide showed a dose and time dependent cytotoxicity. Cancer cell death starts to occur at 48 h with doses as low as 5 nM and an IC-50 ranging from 2 to 5 nM in these cell lines (Fig. 2a). This cell death was associated with an increase in the levels of cleaved caspase-3 at 48 h suggesting that these cells die by apoptosis (Fig. 2b). Since cell lines do not necessarily represent the heterogeneity seen in patients, we evaluated the ability of triptolide on cell proliferation of 17 AML blast samples obtained from patients. At 72 h, all patient derived blasts were sensitive to triptolide with IC-50 ranging from 5 to 9 nM, as demonstrated in Fig. 2c. Thus triptolide shows significant cytotoxic effect in vitro in doses that are much lower compared to the ones traditionally used for solid organ cancers (ranging from 25 to 100 nM) [14].

### Triptolide decreases colony forming ability of AML cancer cells and decreases expression of stem cell markers

**Fig. 3:**
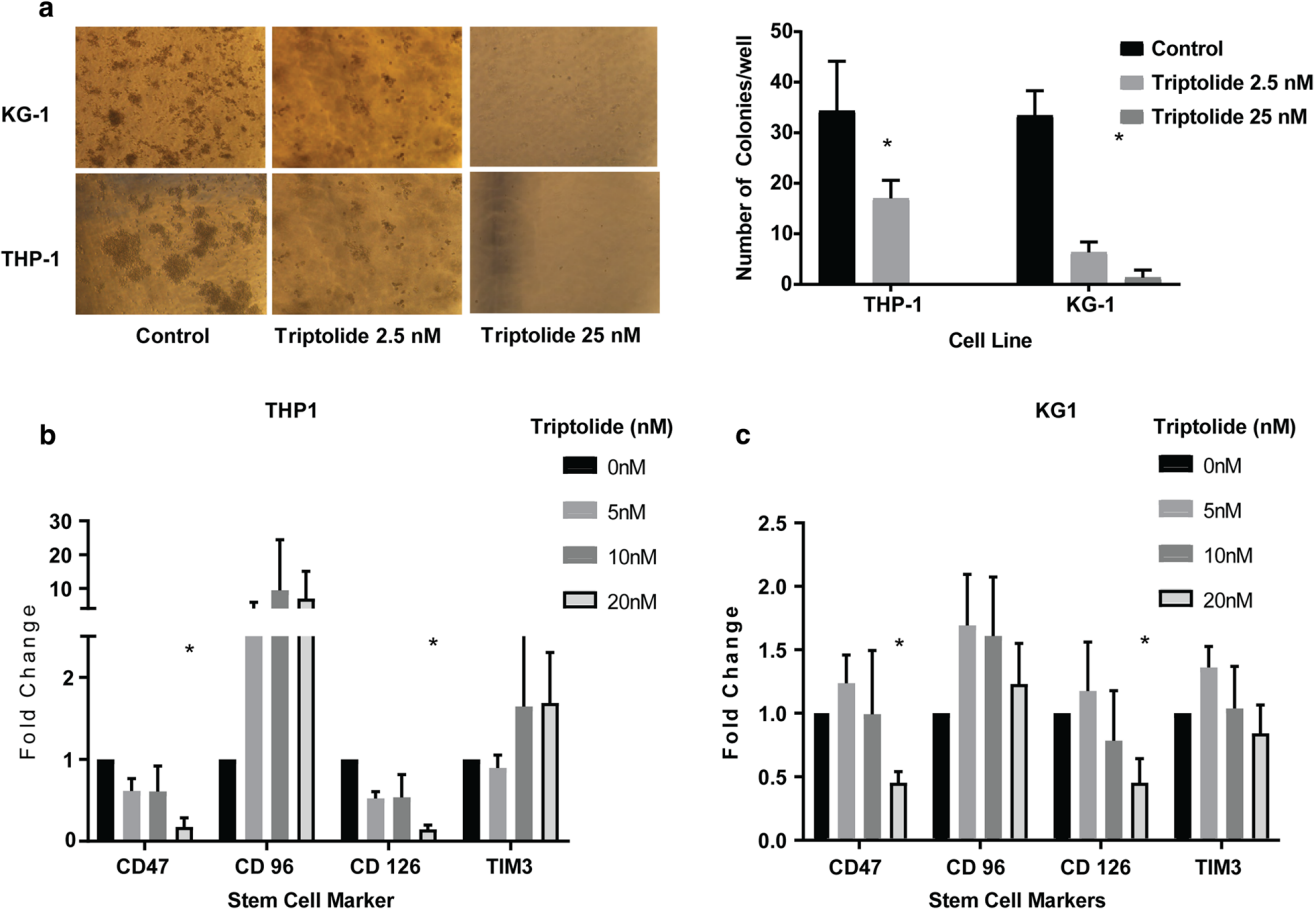
**a** Treatment with triptolide resulted in a decrease in colony forming ability of AML cell lines THP-1 and KG1a as measured by a methylcellulose based colony forming assay. **b**, **c** Treatment with triptolide also caused a decrease in surface markers of commonly expressed stem cell markers in AML cell lines THP-1 (**b**) and KG1a (**c**). n = 3, *p < 0.05 when compared to untreated cells, data shows mean ± SD

Therapy failure and relapse in AML patients is often attributed to the presence of leukemic stem cells [15]. Thus, we measured effect of triptolide on AML stem cells by colony forming assay. Treatment of KG1a and THP-1 cell lines with triptolide (0–25 nM) for 12 h followed by subsequent plating in drug free methylcellulose media resulted in a marked decrease in the number of AML cell colonies suggesting the effect of triptolide on stem cells in AML (Fig. 3a, b). This was further corroborated by a decrease in the mRNA expression of various AML stem cell makers like (CD47, CD34 and CD126) upon treatment of these cancer cells with triptolide for 24 h (Fig. 3c, d).

### Triptolide affects AML cancer cells by downregulating c-Myc and causing cell cycle arrest

**Fig. 4:**
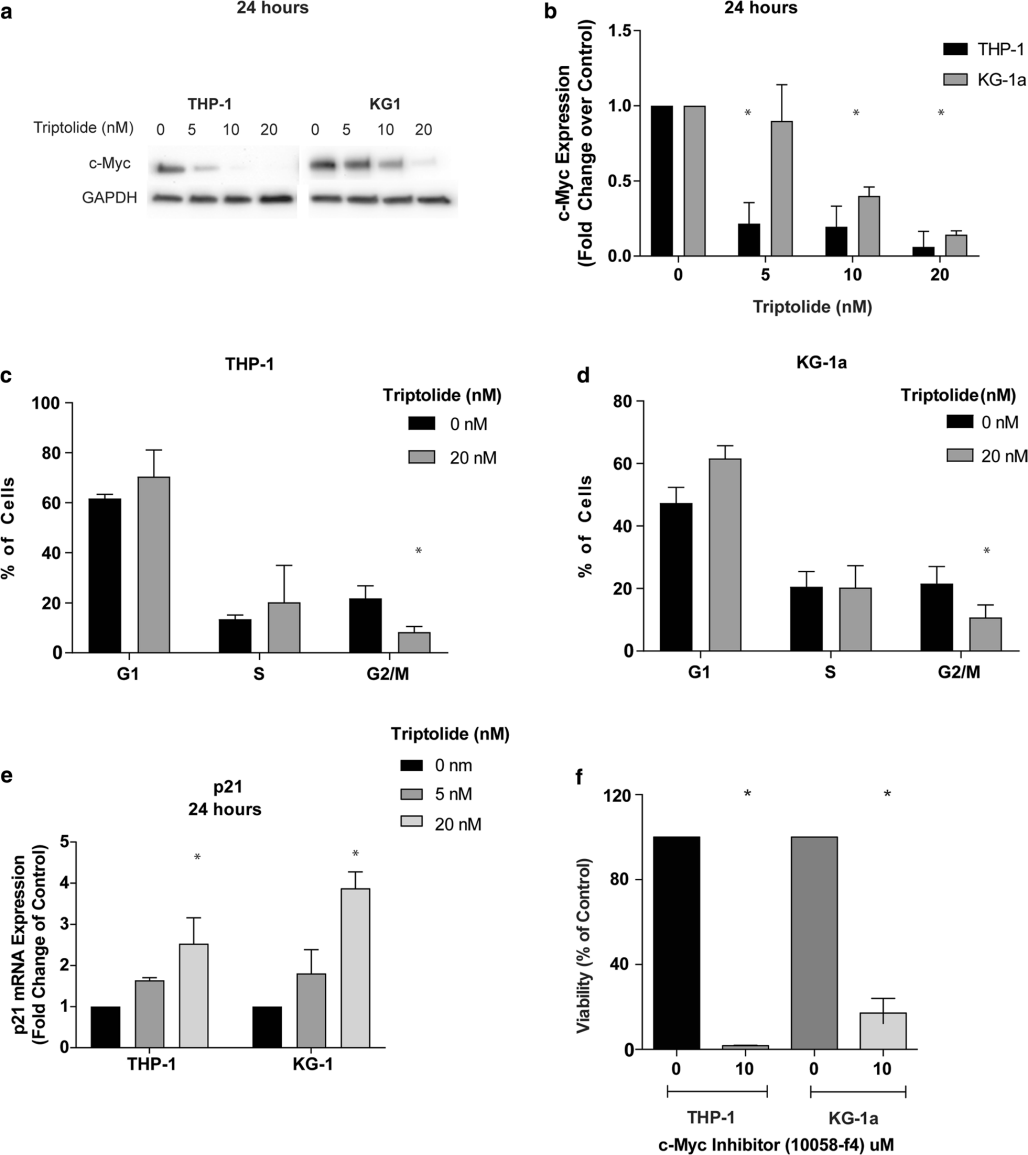
Triptolide caused a decrease in the protein levels of c-Myc in a dose and time dependent manner (**a**). On evaluating mRNA levels, treatment with triptolide reduced c-Myc transcription (**b**). Also, an increase in number of cells at the G1/S interphase with a decrease in G2/M phase of both THP-1 (**c**) and KG1a (**d**) cells were seen with a corresponding increase in the levels of the cell cycle regulator protein p21 with triptolide (**e**). Inhibition of c-Myc using a specific c-Myc inhibitor led to a decrease in cell proliferation (**f**). n = 3, *p < 0.05 compared to control untreated cells, data shows mean ± SD

c-Myc is a transcription factor that is widely dysregulated in AML cells and has been shown to mediate survival of leukemic stem cells [16, 17]. Treatment of AML cells with triptolide resulted in a decrease in the protein levels of c-Myc at 24 h in a dose dependent manner. In THP-1 cells, triptolide at doses as low as 5 nM was effective in decreasing c-Myc levels corresponding to the cytotoxicity that is seen following 24 h of treatment (Fig. 4a). This decrease was due to a reduction in transcription of c-Myc as shown by reduction in the mRNA levels when treated with triptolide in both THP-1 and KG1a cell lines (Fig. 4b).

Since c-Myc regulates cell cycle progression at various stages, but particularly at the G1–S phase [18, 19]. we performed a cell cycle analysis on triptolide treated AML cells. Our results showed that triptolide resulted in an accumulation of cells in the G1–S phase with a decrease in the proportion of cells in the G2/M phase in both cancer cell lines (Fig. 4c, d).

One of the ways c-Myc regulates cell cycle is through the p21 protein (cyclin-dependent inhibitor-1). p21 is a cell cycle inhibitor that is known to be negatively regulated by c-Myc [18]. We find that corresponding with the decrease in c-Myc levels, there is an increase in the expression of p21 in a dose dependent fashion in AML cancer cells (Fig. 4e).

This together suggests that triptolide causes cancer cell death by decreasing the transcription of c-Myc, which in turn results in an increase in the levels of p21 causing cell cycle arrest and ultimately leading to cell death via apoptosis. We further confirmed this finding using a specific inhibitor for c-Myc against AML cancer cells, 10058-F4 (Fig. 4f). Treatment with 10058-F4 resulted in cell death in both THP-1 and KG1a recapitulating the effects seen with triptolide.

## Discussion

Treatment for AML that is effective and also well tolerated has been a focus of intense research for the last six decades [20]. But despite major breakthroughs in the molecular and phenotypic characterization of AML, standard therapy has remained largely unchanged for the last four decades [21, 22]. The prognosis with chemo-intensive regimen of cytarabine and anthracyclines is modest at best in young patients. However, a greater proportion of the patients diagnosed with AML are elderly and present with significant co-morbidity, precluding any hope for long term remission [3]. In this backdrop, there is an unmet need for newer therapeutic modalities to treat AML.

Through our studies, we show that Minnelide is effective against AML in multiple pre-clinical models. Treatment with Minnelide resulted in complete clearance of leukemic burden in AML models of both cell line and patient derived AML blasts. The doses that are able to clear leukemia in these models ranges from 0.1 to 0.15 mg/kg/day. We found that Minnelide, when used for AML, is effective at low doses. These doses are much lower than equivalent human doses that were safely tolerated by patients in the Phase I Clinical Trial of Minnelide [12].

This is confirmed by our findings in vitro, where triptolide, the active metabolite of Minnelide, leads to apoptosis in cancer cells at doses as low as 5 nM. This efficacy is seen over a wide range of AML cancer cell lines and patient blast samples.

Consistent with its cytotoxic effect, treatment with triptolide resulted in a decrease in clonogenicity, a marker of stemness, as well as reduced expression of stem cell markers. More interestingly though, Minnelide when administered at a higher dose of 0.21 mg/kg/day had no effect on normal hematopoietic myeloid precursors, highlighting its selectivity against leukemic cells.

The broad effect of triptolide against leukemic cell lines and patient samples pointed towards triptolide’s (Minnelide) effect against a pathway that is commonly dysregulated in leukemias. One such transcription factor regulating multiple processes in AML is c-Myc. We found that treating AML cells with triptolide resulted in a significant decrease in transcription and ultimately translation of c-Myc. In AML, c-Myc is overexpressed via multiple mechanisms. Myc amplification is described to occur via double minute chromosomes [23, 24] in AML and c-Myc overexpression is a common end result of fusion oncoproteins such as AML/ETO, PML-RARA and PLZF-RARA [25]. Furthermore, activating mutations in the pathways of receptor and non-receptor tyrosine kinase is also shown to dysregulate c-Myc [26]. These mutations can be found in almost 50% of patients with AML [27] and other studies have highlighted its role as a central downstream transcription factor mediating AML progression [28]. Consistent with its role in cell cycle regulation, we saw that inhibition of c-Myc with triptolide caused an arrest in G1–S phase, a progressive decrease in cells in the G2/M phase and an upregulation of the cell cycle checkpoint protein p21. C-Myc is also known to be an important mediator of cell survival in leukemic stem cells [16, 17] and may explain the reduction in colony forming ability as well as the decrease in stemness markers seen on treatment with triptolide. Subsequently, inhibition of c-Myc with a specific chemical inhibitor resulted in cell death of AML cancer cell lines recapitulating the effects seen with triptolide.

In summary, we show that Minnelide results in a dramatic reduction in leukemic burden in various pre-clinical models of AML at doses that can be easily achieved clinically in patients. In vitro, we see that triptolide, the active component of Minnelide, leads to inhibition of c-Myc transcription in AML cells which subsequently causes cell cycle arrest and cell death via apoptosis. Triptolide is also effective in decreasing the “stemness” of AML cells contributing to the long-lasting remission seen on stopping drug treatment.

## Conclusion

Here we show that Minnelide is effective in decreasing leukemic burden in AML through multiple pre-clinical models and at doses that are clinically applicable. In vitro, we show that triptolide, the active metabolite of Minnelide, results in cell cycle arrest, cell death and reduced clonogenicity of leukemic blasts. Evaluation of Minnelide in patients with AML holds great promise in this difficult disease.

## Materials and methods

### Cell culture and drugs

THP-1, HL-60, Kasumi-1 and KG1a cell lines were obtained from the ATCC and grown in RPMI-1640 with varying concentrations of FBS (10%, 10%, 20% and 20% respectively) with 1% penicillin streptomycin. Luciferase expressing THP-1 were a gift by Dr. Largeaspada from the University of Minnesota. These cell lines were grown under standard conditions at 37 °C in a humidified atmosphere with 5% CO_2_. All cell culture media were obtained from Invitrogen Life Technologies (MA). To make a stock concentration, Triptolide (EMD Millipore, MA) was first diluted in dimethyl sulfoxide (DMSO) to obtain a 12.4 mM stock, stored at − 20 °C and further diluted to indicated concentrations. 10058-F4 (Catalog No: F3680 Millipore Sigma, MA, US) was diluted at a concentration of 1000 µM in DMSO and used at indicated doses.

### Determination of cell viability and apoptosis

Cell Titer Glo (Catalog Number: G755A, Promega, WI) was used as per manufacturer’s directions to evaluate cell viability after treatment of AML cells in 96 well white opaque plates for the indicated time duration and drug concentrations. Apoptosis was measured using caspase 3 Glo assay (Catalog No: G8090, Promega, WI) as described by the manufacturer’s protocol. Briefly, leukemia cells were simultaneously assessed for cell viability as well as caspase 3/7 activity using a pro-luminescent caspase − 3/7 substrate which is cleaved by the caspase 3/7 enzyme and generates a glow type luminescent signal proportional to the caspase 3/7 activity. All experiments were done in triplicates and repeated three independent times.

### Flow cytometric analysis of cell cycle

THP-1 and KG1a cells were grown at a concentration of 5 × 10^6^ cells/ml in 5% RPMI for 24 h followed by changing to serum free media overnight. These cells were then treated with triptolide and harvested after 24 h of treatment. After fixing the cells with 70% ethanol, the leukemic cells were subsequently stained with Guava Cell Cycle Reagent for Flow Cytometry (Cat. No. 4500-0220, EMD Millipore, Billerica, MA) and analysed in BD FACSCanto II (BD Biosciences, CA) BD FACS Canto II (BD Biosciences, CA) using FACS Diva and FlowJo (Tree Star) software.

### Colony forming assays

THP-1 and KG1a cells were treated overnight with indicated concentrations of triptolide and were subsequently washed and re-suspended in Human Methylcellulose complete media (Catalog Number: HSC003, R&D Systems, MN, US) in 24 well plates. Cluster of cells consisting of more than 40 cells were counted as colonies and were scored for number of colonies.

### Quantitative real-time polymerase chain reaction and western blot

THP-1 and KG1a cells (5 × 10^5^ cells/ml) were seeded in 6 well plates and treated with triptolide. These cells were then harvested after washing with PBS (phosphate buffered saline) and RNA was extracted using TRIzol (Life Technologies, MA) reagent according to the manufacturer’s instructions. To make cDNA, total RNA of 2 μg was used with the high capacity cDNA reverse transcription kit (Applied Biosystems, NY). The QuantiTect SYBR Green PCR kit (Qiagen, CA) was used according to the manufacturer’s instructions to run quantitative real-time PCR. Individual values of the genes were expressed relatively by normalizing to the housekeeping gene 18S. Primers were obtained from Integrated DNA Technologies Inc (IA) and are detailed in Additional file 3: Table S1.

For western blotting, AML cells after treatment with indicated drugs and concentrations were washed with PBS once and cell lysates were prepared by adding Rapid Immuno Precipitation Assay Buffer (Boston Bioproducts, MA) with protease (Catalog Number: A32963, Thermo Scientific, MA) (20 μl/ml of lysis buffer) and phosphatase inhibitor (Catalog Number: 78420, Thermo Scientific, MA) in ice. The lysates thus obtained were quantified using Pierce bicinchoninic acid protein estimation kit (Catalog Number: 23250, Thermo Scientific, MA) and electrophoresis was performed on sodium dodecyl sulfate-polyacrylamide gel electrophoresis (SDS-PAGE) gels by loading 30–50 μg of protein. The proteins were then transferred to a polyvinylidene difluoride (PVDF) membrane (Catalog Number: 1620177 BioRad, CA) and probed using the following antibodies c-Myc (Catalog Number: 13987, Cell Signaling Inc, MA) and glyceraldehyde 3-phosphate dehydrogenase (GAPDH) (Catalog Number: AB2302, EMD Millipore Inc., Germany) was used as the loading control.

### In vivo experiments

NRGS Mice (NOD.Cg-Rag1^tm1Mom^ Il2rg^tm1Wjl^ Tg(CMV-IL3, CSF2, KITLG)1Eav/J) are NOD Rag 1 γc null mice that over express human IL-3 and GM-CSF. These mice were sub-lethally irradiated using 250 cGy of irradiation using an X ray irradiator and were injected with 2 × 10^6^ leukemic cells via the tail vein on the same day. In another experiment, mice injected with Luciferase expressing THP-1 cells were serially followed for engraftment using In Vivo Imaging System. d-Luciferin (LUCK-1G, Gold Biotechnology Inc., MO) was prepared at a dose of 15 mg/ml and injected intraperitoneally at 10 μl/g of mouse body weight for measuring bioluminescence. IVIS Spectrum (PerkinElmer, MA, US) was used to measure luminescence followed by image quantitation and analysis using the Living Image Software (PerkinElmer, MA, US). For primary human AML blasts, NRGS mice were followed at various intervals for engraftment of leukemic cells by evaluating the level of human CD45+ (Catalog Number 347723, BD Biosciences) leucocytes in the peripheral blood. Briefly, peripheral blood was evaluated after red blood cell (RBC) lysis using ammonium–chloride–potassium (ACK) buffer. Live cells were stained with Anti hCD45 fluorescein isothiocyanate (FITC) and analyzed by flow cytometry. Animals expressing more than 2% of human CD45 were considered to have positive engraftment. Minnelide, dissolved in saline, was administered intra-peritoneally (i.p) at a dose of 0.05 mg/kg/day to 0.1 mg/kg/day.

### Statistical analysis

In the corresponding figures, values are expressed as mean ± SD for in vitro experiments and mean ± SEM for in vivo experiments. For individual experiments, n and p values are mentioned in the corresponding figure legends. Survival was measured using Kaplan–Meier survival curve using Graph pad Prism software (GraphPad Software, inc, California). Data was analyzed using the Mann–Whitney unpaired test for in vivo experiments and in vitro experiments were analyzed with student t-test with two-sided p < 0.05 being considered significant.

## Additional files

**Additional file 1: Figure S1.** Minnelide treated mice have leukemic burden comparable to that of naive background non-leukemic mice.

**Additional file 2: Figure S2.** Minnelide treated mice did not result in significant change in absolute counts or proportion of neutrophils.

**Additional file 3: Table S1.** Primer sequences.

## Abbreviations

ACK: ammonium–chloride–potassium
AML: acute myeloid leukemia
FITC: fluorescein isothiocyanate
GAPDH: glyceraldehyde 3-phosphate dehydrogenase
GI: gastrointestinal
IACUC: International Animal Care and Use Committee
I.p: intraperitoneal
IVIS: In Vivo Imaging System
MTD: maximum tolerated dose
MTT: 3-(4,5-dimethylthiazol-2-yl)-2,5-diphenyl tetrazolium bromide
NRG: NOD.Rag1−/−;γcnull
NRGS: NRG-SGM3
PBS: phosphate buffered saline
PVDF: polyvinylidene difluoride
RBC: red blood cell
ROI: region of interest
SDS-PAGE: sodium dodecyl sulfate-polyacrylamide gel electrophoresis

## Availability of data and materials

All data generated or analyzed during this study are included in this published article [and its additional information files].
