# Correction to: Pre-clinical evaluation of Minnelide as a therapy for acute myeloid leukemia

**DOI:** 10.1186/s12967-019-2048-3

**Published:** 2019-09-04

**Authors:** Bhuwan Giri, Vineet K. Gupta, Brianna Yaffe, Shrey Modi, Pooja Roy, Vrishketan Sethi, Shweta P. Lavania, Selwyn M. Vickers, Vikas Dudeja, Sulagna Banerjee, Justin Watts, Ashok Saluja

**Affiliations:** Sylvester Comprehensive Cancer Center and DeWitt Daughtry Family Department of Surgery, University of Miami, 460C CRB Research Building, 1140 NW 14th St, Miami, FL 33136 USA

## Correction to: J Transl Med (2019) 17:163 10.1186/s12967-019-1901-8

Following publication of the original article [1], the authors found an error in Fig. 3. The middle panel of Fig. 3a was inadvertently duplicated.

In this Correction the incorrect and corrected version of Fig. 3 are shown.

Originally Fig. 3 was published as:

**Fig. 3:**
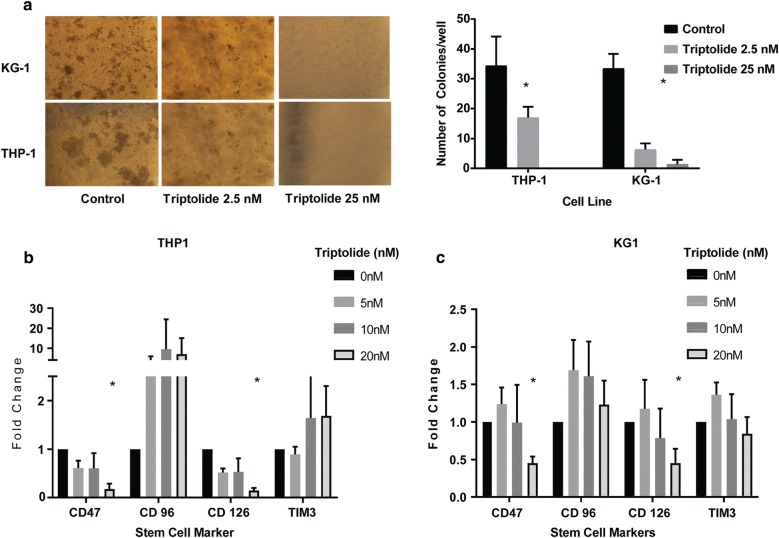
**a** Treatment with triptolide resulted in a decrease in colony forming ability of AML cell lines THP-1 and KG1a as measured by a methylcellulose based colony forming assay. Treatment with triptolide also caused a decrease in surface markers of commonly expressed stem cell markers in AML cell lines THP-1 (**b**) and KG1a (**c**). n = 3, *p < 0.05 when compared to untreated cells, data shows mean ± SD

The corrected version of Fig. 3:

**Fig. 3:**
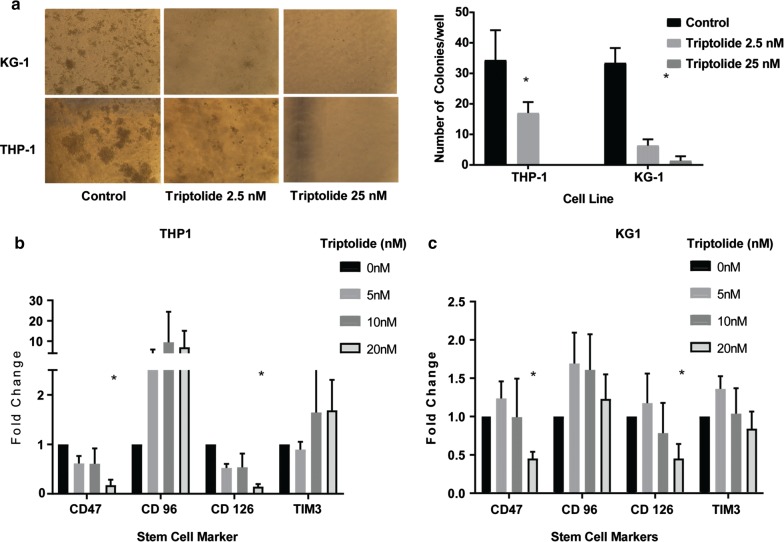
**a** Treatment with triptolide resulted in a decrease in colony forming ability of AML cell lines THP-1 and KG1a as measured by a methylcellulose based colony forming assay. Treatment with triptolide also caused a decrease in surface markers of commonly expressed stem cell markers in AML cell lines THP-1 (**b**) and KG1a (**c**). n = 3, *p < 0.05 when compared to untreated cells, data shows mean ± SD
