# Supplementary figures and images for: Pre-clinical evaluation of Minnelide as a therapy for acute myeloid leukemia
Source: J Transl Med. 2019 May 20;17:163. doi: 10.1186/s12967-019-1901-8 (PMC6528210; doi:10.1186/s12967-019-1901-8)

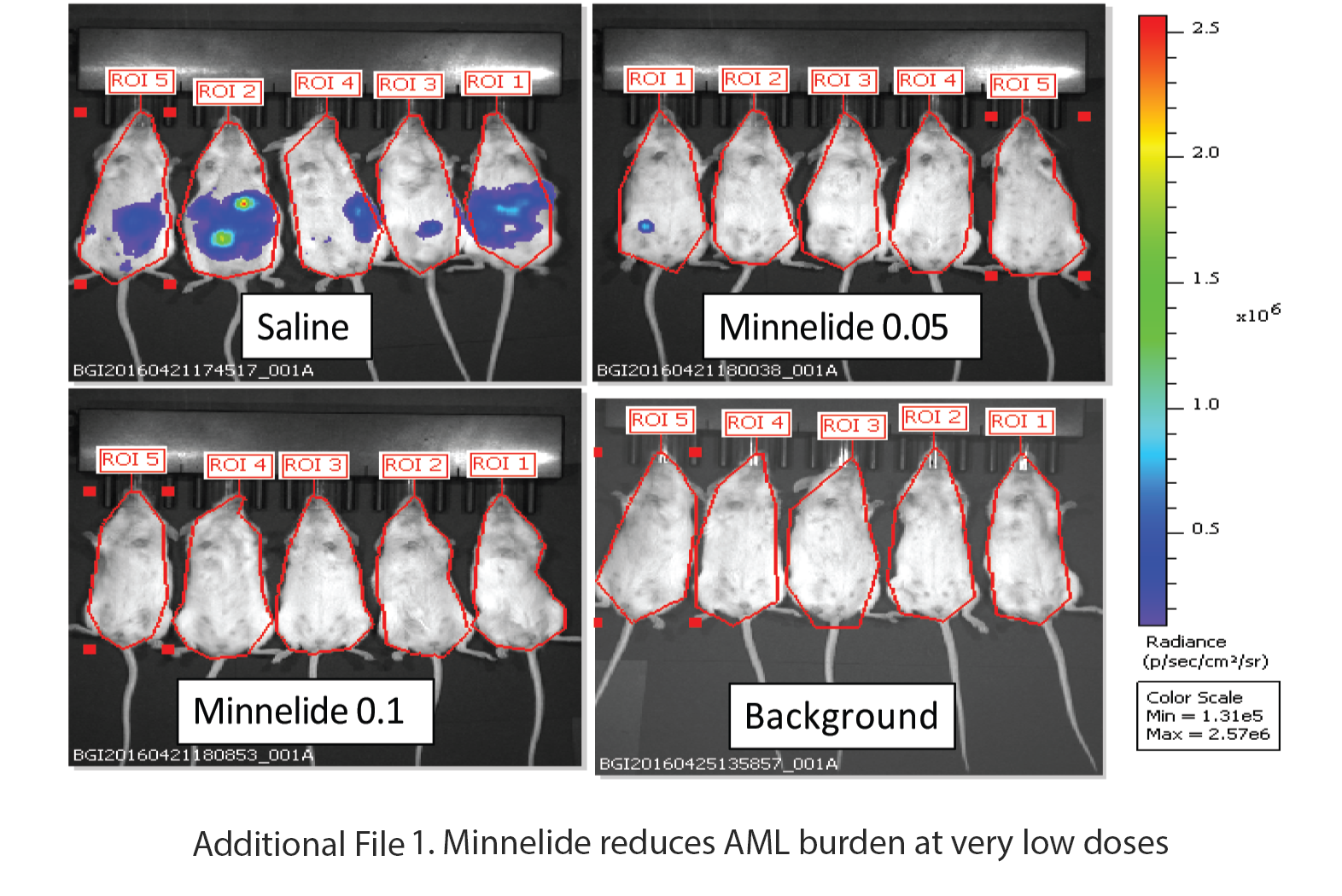

Supplement: Supplementary file 1 — Additional file 1: Figure S1. Minnelide treated mice have leukemic burden comparable to that of naive background non-leukemic mice. [file 12967_2019_1901_MOESM1_ESM.tif]

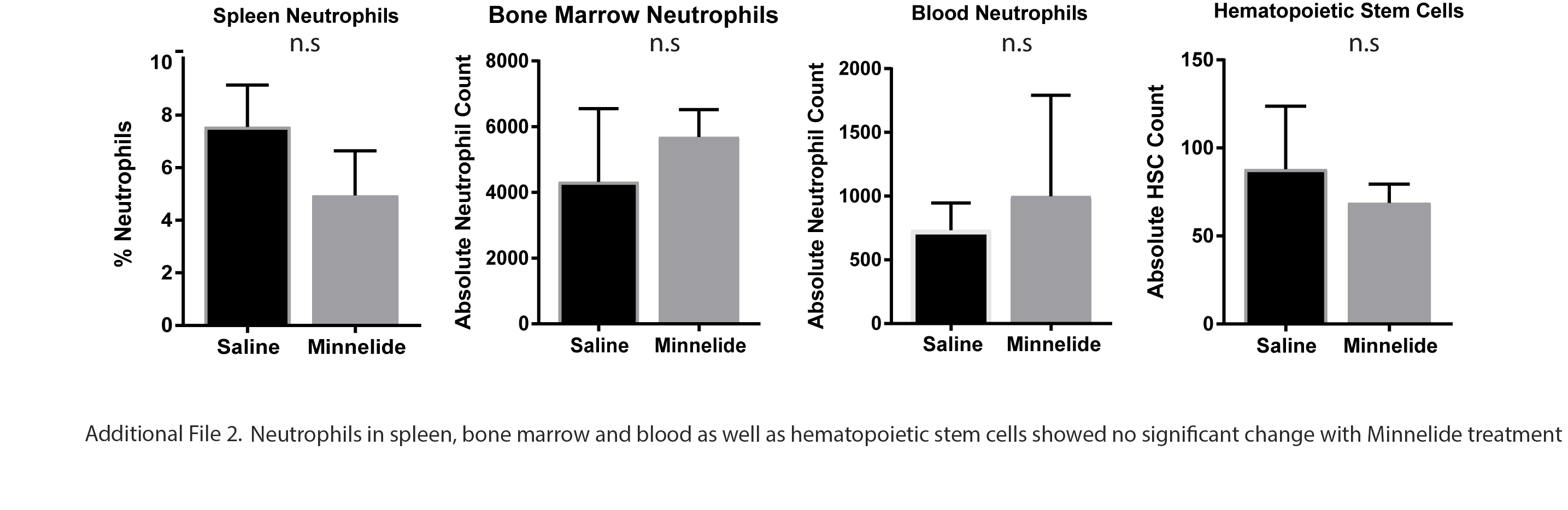

Supplement: Supplementary file 2 — Additional file 2: Figure S2. Minnelide treated mice did not result in significant change in absolute counts or proportion of neutrophils. [file 12967_2019_1901_MOESM2_ESM.tif]
