# Supplementary material for: Pre-clinical evaluation of Minnelide as a therapy for acute myeloid leukemia
Source: J Transl Med. 2019 May 20;17:163. doi: 10.1186/s12967-019-1901-8 (PMC6528210; doi:10.1186/s12967-019-1901-8)
Supplement: Supplementary file 3 — Additional file 3: Table S1. Primer sequences. [file 12967_2019_1901_MOESM3_ESM.docx]

| ***Gene*** | ***Forward Primer*** | ***Reverse Primer*** | ***Product length*** |
| --- | --- | --- | --- |
| *CD47* | *CCAAAAGACCCTGATTGCAC* | *TAAGGGTCTTCGCCCAGC* | *196* |
| *CD 96* | *TCCTGTCTACGTTTCATTTCCT* | *CATCAGAGCCAAGTGTAGCA* | *290* |
| *CD 126* | *CTTCTTCAGTACCACTGCCC* | *GTGGCTCGAGGTATTGTCAG* | *262* |
| *TIM3* | *TCAGAATGCCTATCTGCCCT* | *CGTTGCCACATTCAAACACA* | *104* |

**Additional file 3: Table S1: Primer sequences**
